# TyG–BMI as a Marker of Metabolic Status and Its Association with Bone Quality and Mineral Metabolism: A Sex-Specific Analysis

**DOI:** 10.3390/jcm15114226

**Published:** 2026-05-29

**Authors:** Assel Anarbayeva, Karlygash Sadykova, Gulnaz Nuskabayeva, Nursultan Nurdinov, Nurgul Zholdassova, Dilfuza Mamraimova, Gulzira Baimakhanova, Dinara Azizkhojayeva, Sirozhiddin Irismetov, Shoira Isanova, Ugilzhan Tatykayeva

**Affiliations:** 1 Department of Special Clinical Disciplines, Faculty of Medicine, Khoja Akhmet Yassawi International Kazakh-Turkish University, Turkestan 161200, Kazakhstan; 2 Department of Fundamental Medical Sciences, Faculty of Dentistry, Khoja Akhmet Yassawi International Kazakh-Turkish University, Turkestan 161200, Kazakhstan; 3 Department of Neurology, Faculty of Medicine, Samarkand State Medical University, Samarkand 140100, Uzbekistan

**Keywords:** TyG–BMI, insulin resistance, bone quality, quantitative ultrasound, mineral metabolism, sex differences

## Abstract

**Background:** Insulin resistance (IR) is a major pathogenic factor in metabolic syndrome and related diseases. It is challenging to accurately measure IR due to high cost and technical complexity. TyG-derived indices have been proposed as simple and reliable surrogates for assessing insulin resistance. The present study aimed to investigate the association between TyG-derived indices and indicators of mineral metabolism and bone quality and to examine sex-specific differences. **Methods:** This cross-sectional study included 330 adults aged 18–65 years. Age-adjusted partial correlation analyses and multivariable linear regression models were performed to examine the associations between TyG indices and several metabolic parameters, minerals, and QUS bone indices. **Results:** TyG–BMI was strongly correlated with triglycerides (females: r = 0.51; males: r = 0.54, both *p* < 0.001), glucose (females: r = 0.54, *p* < 0.001), and hip circumference (females: r = 0.77; males: r = 0.58, both *p* < 0.001). HDL showed inverse associations (females: r = −0.41, *p* < 0.001). In females, TyG–BMI demonstrated modest positive correlations with bone parameters, including BQI (r = 0.21, *p* = 0.002), T-score and Z-score (r = 0.21, *p* = 0.002 for both), SOS (r = 0.19, *p* = 0.007), and BUA (r = 0.18, *p* = 0.010), whereas no significant associations were observed in males. In multivariable models, TyG–BMI in females remained independently associated with glucose (β = 9.77, *p* < 0.001), waist circumference (β = 2.94, *p* < 0.001), and HDL (β = −26.6, *p* < 0.001), but not with mineral or bone parameters. **Conclusions:** The TyG-derived indices, most notably TyG–BMI, are useful indicators of metabolic status that correlate with bone quality yet exhibit sex-specific associations. These findings support the role of TyG-derived indices as accessible surrogate markers of metabolic status and demonstrate sex-specific associations with bone quality parameters.

## 1. Introduction

The incidence of metabolic disorders such as obesity, insulin resistance, and type 2 diabetes mellitus is rapidly increasing over the past few decades, and it has become one of the main public health challenges of the 21st century [1]. Insulin resistance (IR) is the core pathophysiological feature underlying common diseases with serious adverse cardiometabolic risks. The hyperinsulinemic-euglycemic clamp is currently considered the gold standard for assessment of insulin resistance, but it is not practical for large-scale or clinical use due to its labor-intensive protocol, need for specialized equipment, and time consumption [2]. Moreover, surrogate markers of insulin resistance such as Homeostatic Model Assessment of Insulin Resistance (HOMA-IR) require a measurement of insulin that is expensive and not always readily available [3].

The triglyceride–glucose (TyG) index is a simple, reliable, and widely available biochemical surrogate marker for insulin resistance [4]. The additional TyG-derived indices, such as TyG–BMI (biochemical IR and body mass index) and TyG–WC (biochemical IR and waist circumference), have been proposed for improving the prediction of metabolic syndrome and related diseases [5,6,7]. TyG–BMI combines information on lipid and glucose metabolism with overall adiposity, reflecting both systemic and structural insulin resistance. Additional studies have demonstrated that these indices outperform TyG in the prediction of metabolic syndrome, NAFLD, and cardiovascular diseases (CVD) [8,9]. A recent study of Chinese women with PCOS showed that high TyG–BMI was a strong predictor for insulin resistance (OR = 2.75, *p* < 0.001; AUC = 0.841, *p* < 0.001) and metabolic syndrome (OR = 4.18, *p* < 0.001; AUC = 0.899, *p* < 0.001). TyG–BMI appears to be a simple, cost-effective, and clinically relevant surrogate marker for assessment of metabolic dysfunction in different populations.

In addition to metabolic disturbances, abnormal levels of several minerals and their related endogenous and dietary indices are found to characterize the insulin-resistant state [10,11]. These minerals play critical roles in both insulin action and bone remodeling. Moreover, their combination in derived mineral indices may provide a further level of insight into the pathophysiology of insulin resistance. For example, an elevated Ca/Mg ratio may characterize insulin resistance [12], while low vitamin D (VitD)/parathyroid hormone (PTH) indicate secondary hyperparathyroidism, which worsens IR [13].

Moreover, metabolic disturbances associated with insulin resistance can affect skeletal health [14,15]. Assessing skeletal health is typically made by calculating T and Z-scores, which describe the deviation of bone mineral density (BMD) from a young reference population and age-matched controls, respectively. However, these parameters do not necessarily reflect changes in bone microarchitecture that may have occurred as a result of a metabolically compromised state [15]. An alternative method for the assessment of skeletal health is provided by quantitative ultrasound (QUS) using instruments that measure speed of sound (SOS) and broadband ultrasound attenuation (BUA). These parameters were incorporated into the bone quality index (BQI). Importantly, patients with insulin resistance and metabolic syndrome can have normal or even increased BMD but impaired bone quality, which requires more sensitive methods for its assessment, such as QUS [16,17]. Combining these TyG-derived metabolic indices with advanced bone quality assessment can provide a better understanding of the metabolic–bone axis.

Despite the growing interest in TyG-derived indices, data on the relationship between TyG and indicators of bone quality in the Central Asian population, including Kazakhstan, are currently limited. Given widespread vitamin D deficiency [18] and the high prevalence of metabolic disorders [19] in the Republic of Kazakhstan, it is very important to study the connection between TyG and indicators of bone quality in both sexes. This study aims to investigate the correlation between indices of TyG with indices of mineral metabolism and bone quality and to explore the sex-specific differences between them.

## 2. Materials and Methods

### 2.1. Study Design and Population

This is an analytical cross-sectional study conducted among adults who attended regular doctor appointments at the Clinical Diagnostic Center of the International Kazakh–Turkish University, named after Khoja Ahmed Yassawi, between January and December of 2025.

Inclusion criteria for study participants were adults 18–65 years of age who provided written informed consent to participate. Exclusion criteria for this study were acute or recent (within the last 3 months) infectious illness, presence of a chronic condition that requires immediate medical intervention (e.g., severe injuries, life threatening illnesses, unstable medical conditions), presence of any psychiatric disorder, pregnant or lactating women (including women in the postpartum period up to 6 months after delivery), presence of secondary osteoporosis (e.g., endocrine, autoimmune, or other medical illnesses that cause bone loss), history of cancer, diagnosed chronic kidney disease, severe hepatic or cardiovascular disease, use of medications known to affect mineral metabolism within the last 30 days (e.g., glucocorticoids, high doses of calcium, magnesium, or vitamin D), history of calcaneal fractures or implants in the measurement area, heavy alcohol use, and morbid obesity (BMI > 40 kg/m^2^). A total of 330 patients consented to participate.

### 2.2. Clinical and Anthropometric Assessment

All participants were assessed in a clinical setting using standard experimental protocols that included assessment of participants’ medical history, physical assessment, and measurement of blood, systolic and diastolic blood pressure, and heart rate. Anthropometric parameters such as height, weight, waist (at the level of the umbilicus), hips, and body mass index (BMI in kg/m^2^) were recorded.

### 2.3. Laboratory Measurements

Samples of venous blood were collected in the morning after an overnight fast of 8–12 h. Biochemical analyses were conducted on these samples in a certified clinical laboratory using standard analytical methods.

The following parameters were measured using enzymatic colorimetric methods (enzymatic kits) (Roche Cobas, Penzberg, Germany) on an automated analyzer: total cholesterol, triglycerides, LDL-C, HDL-C. Magnesium in serum and Inorganic Phosphorus were measured by colorimetric methods with an automated analyzer. Fasting plasma glucose was estimated using the hexokinase method. Glycated hemoglobin (HbA1c) was estimated using an immunoturbidimetric assay, which complies with NGSP/IFCC standards.

Ionized calcium was determined using an ion-selective electrode. The serum concentrations of VitD—25-hydroxy vitamin D (25(OH)D)—and PTH were determined using an electrochemiluminescence immunoassay. All analyses were performed following quality control rules, which are in accordance with the international standards.

### 2.4. TyG-Derived Indices and Mineral Metabolism Indices

The triglyceride–glucose (TyG) index was calculated using the standard formula, where triglycerides and glucose were expressed in mg/dL:

$$TyG=\ln\left(\frac{TG\times Glucose}{2}\right)$$

The indices used as surrogate markers of IR were calculated based on the following formulas:

TyG–BMI = TyG × BMI

TyG–WC = TyG × WC

In addition to the single mineral metabolism parameters such as calcium (Ca), magnesium (Mg), phosphate (P), parathyroid hormone (PTH), and 25-hydroxy vitamin D (25(OH)D), several derived variables of the mentioned parameters were calculated to possibly recognize interactions of the individual variables. The variables calculated were the Ca/Mg ratio, the product of Ca and P (Ca × P), the P/Mg ratio, and the Ca × P to Mg ratio. Furthermore, the ratio of 25(OH)D to PTH (VitD/PTH) was calculated as an exploratory variable to gain a perception of the dynamic of the vitamin D-PTH axis.

### 2.5. Bone Assessment (Quantitative Ultrasound)

QUS densitometry of the calcaneus was performed to assess skeletal health. SOS in m/s is an indicator of bone density and stiffness, and the BUA in dB/MHz reflects trabecular architecture. The BQI is a bone strength indicator that can be obtained from SOS and BUA. In addition, a T-score, which is an indicator of bone loss relative to a young, healthy population, and a Z-score, which is an indicator of bone health relative to age-matched controls in the reference database of the device, were reported. All measurements were performed using the SONOST 3000 (OsteoSys, Seoul, Republic of Korea).

### 2.6. Ethical Approval

The study protocol was approved by the Ethics Committee of the International Kazakh–Turkish University, named after Khoja Ahmed Yassawi. Participants gave their informed consent. The present study was conducted according to the principles expressed in the Declaration of Helsinki and the study protocol was approved by the Ethics Committee on 24 October 2024 (№ 34).

### 2.7. Statistical Analysis

For descriptive statistics, continuous variables are presented as means (±standard deviation) and categorical variables as frequencies (percentages). Normality of continuous variables was assessed using the Shapiro–Wilk test together with visual inspection of histograms and Q–Q plots. Homogeneity of variances between groups was evaluated using Levene’s test. Student’s *t*-test and Pearson’s chi-squared test were used to compare the male and female groups in terms of continuous and categorical variables, respectively. The non-parametric tests were not used since all assumptions for the parametric tests were met. There were no missing data for the laboratory, anthropometric, or QUS variables included in the study.

To clarify the association between TyG-derived indices and metabolic parameters, mineral metabolism markers, and bone indices, partial correlation analyses were performed after adjusting for age. To identify variables that are independently associated with TyG–BMI, multivariable linear regression analyses were performed for males and females separately. All potentially related variables were included in the univariate analysis to select relevant variables for the multiple linear regression models. Statistically significant variables were included in the adjusted models one by one, and partial F-tests were performed to assess the additional contribution of each variable to the goodness-of-fit of the model. Variance inflation factors (VIF) for all variables and the whole model were determined to assess the level of multicollinearity. Values less than 5 are generally considered satisfactory. A two-sided *p*-value less than 0.05 was considered statistically significant. All analyses were performed using STATA 16.0.

## 3. Results

The demographic information for the participants is summarized in Table 1. The 330 participants included in the study had a mean age of 46 (±12) years and included 231 (70%) females. There was no gender difference in the mean age of the participants. However, significant sex differences existed in some clinical and metabolic indexes. Men had significantly higher levels of insulin resistance indices than women, including TyG–BMI [260.5 (±48.7) vs. 242.2 (±55.7), *p* = 0.007] and TyG–WC [916.3 (±141.1) vs. 795.9 (±142.2), *p* < 0.001]. A similar tendency was observed for triglyceride levels.

Compared to female lipoprotein levels, the male lipid profile revealed increased levels of LDL cholesterol (*p* = 0.019) and non-HDL cholesterol (*p* = 0.005). Conversely, male HDL cholesterol was decreased compared to female HDL cholesterol: 1.05 (±0.20) vs. 1.35 (±0.33) mmol/L, *p* < 0.001. The levels of total cholesterol were not different between the genders.

Indicators of glycemic status were elevated in males, with fasting glucose being 6.04 (±2.59) mmol/L compared with the controls at 5.25 (±1.82) mmol/L (*p* = 0.003), and HbA1c being 5.89 (±1.80)% compared with 5.32 (±1.78with *p* = 0.011.

Table 2 shows the distribution of mineral metabolism markers and bone strength (QUS) parameters by gender among the study participants. Although most individual biochemical parameters did not show any significant differences between females and males, some of them had evident tendencies. In the mineral metabolism, magnesium was significantly higher in males: 0.83 (±0.07) vs. 0.80 (±0.06) mmol/L with *p* = 0.018. Several of the mineral indices showed sex-specific differences. Female participants had significantly greater Ca/Mg, P/Mg, and composite Ca × P/Mg ratios, but the Ca × P and VitD/PTH products were not different.

Significant sex differences were found in bone strength assessed by QUS. Mean T-scores for males were significantly higher than those for females: 83.2 (±16.9) and 74.5 (±13.2), respectively. For Z-scores, values were significantly higher in males than in females: 95.4 (±21.1) vs. 86.1 (±15.9) with *p* < 0.001. However, the distribution of cases into categorical groups based on Z-score did not show a significant difference.

In men, the mean SOS was significantly higher than in women (1516.5 ± 17.9 vs. 1508.5 ± 11.4, *p* < 0.001). In addition, the mean BQI was significantly higher in men (86.5 ± 17.5 vs. 77.6 ± 13.7, *p* < 0.001). A similar tendency was observed for BUA (*p* = 0.024).

Table 3 shows age-adjusted partial correlations between TyG–BMI and TyG–WC with metabolic parameters by sex. Triglycerides showed a positive correlation with both TyG-derived indices in females (r = 0.51, *p* < 0.001 and r = 0.57, *p* < 0.001, respectively) and males (r = 0.54 with *p* < 0.001 for both). HDL cholesterol showed a negative correlation with TyG–BMI and TyG–WC in both genders, and the correlation in females (r = −0.41 and *p* < 0.001 for both) was more significant than that in males (r = −0.24, *p* = 0.027 and r = −0.32, *p* = 0.002, respectively).

Correlations between glucose (r = 0.54) and HbA1c (r = 0.44) with TyG–BMI were moderate and significant in females (*p* < 0.001 for both), while in males, correlations were weaker and not significant. No correlation was found between LDL, total cholesterol, and TyG–BMI. However, there were positive correlations between hip circumference and both TyG indices in both genders (r = 0.58–0.77, *p* < 0.001).

**Figure 1:**
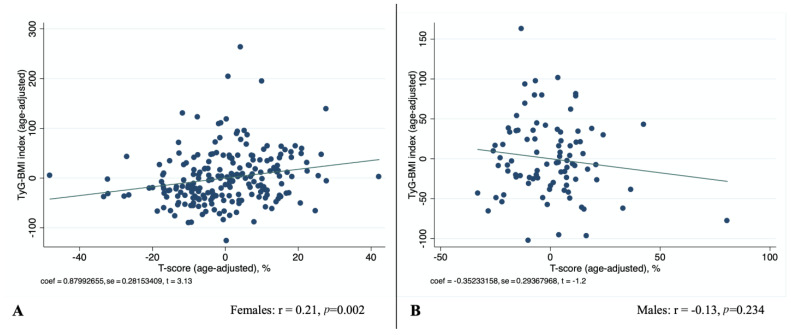
Age-adjusted association between TyG–BMI and T-score stratified by sex: (**A**) females; (**B**) males.

**Figure 2:**
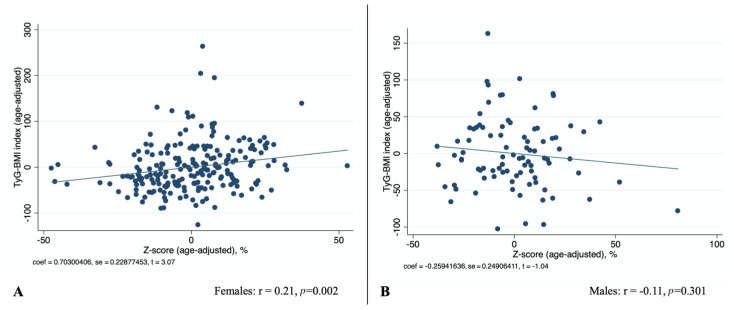
Age-adjusted association between TyG–BMI and Z-score stratified by sex: (**A**) females; (**B**) males.

**Figure 3:**
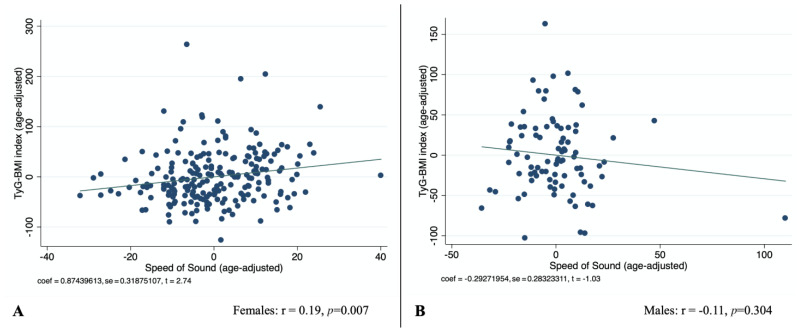
Age-adjusted association between TyG–BMI and SOS stratified by sex: (**A**) females; (**B**) males.

**Figure 4:**
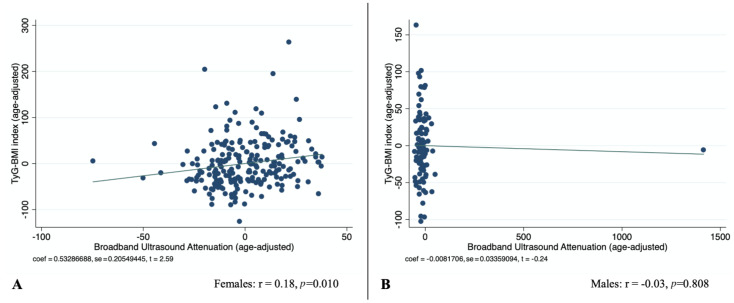
Age-adjusted association between TyG–BMI and BUA stratified by sex: (**A**) females; (**B**) males.

**Figure 5:**
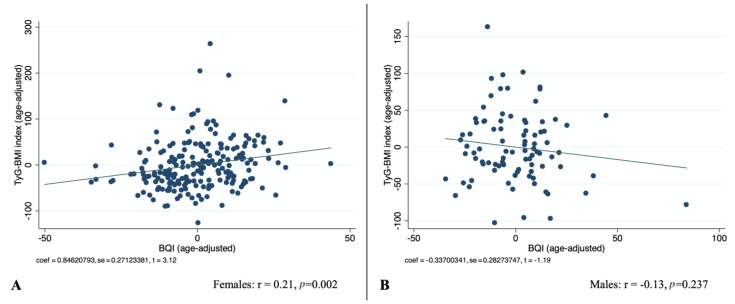
Age-adjusted association between TyG–BMI and BQI stratified by sex: (**A**) females; (**B**) males.

Age-adjusted associations between TyG–BMI and bone strength parameters (BQI, T-score, Z-score, SOS, and BUA) by sex are shown in Figure 1, Figure 2, Figure 3, Figure 4 and Figure 5.

In females, TyG–BMI showed modest but significant positive correlations with all bone parameters, including T-score (r = 0.21, *p* = 0.002), shown in Figure 1; Z-score (r = 0.21, *p* = 0.002), shown in Figure 2; SOS (r = 0.19, *p* = 0.007), shown in Figure 3; BUA (r = 0.18, *p* = 0.010), shown in Figure 4; and BQI (r = 0.21, *p* = 0.002), shown in Figure 5. In males, all corresponding associations were negative but not significant (r from −0.03 to −0.13, all *p* > 0.05).

Supplementary Table S1 shows all mineral metabolism parameters and their age-adjusted correlation with TyG–BMI and TyG–WC. Although many additional significant correlations were found, they were not depicted in the figures. Of note, in females, TyG–BMI had a weak correlation with the VitD/PTH ratio (r = −0.15, *p* = 0.029). TyG–BMI and TyG–WC were positively correlated with all bone parameters (T-score, Z-score, BQI, SOS, and BUA).

Compared with female participants, fewer significant correlations were found in the male group. Within the male group, TyG–WC was negatively associated with T-score (r = −0.22, *p* = 0.037), Z-score (r = −0.21, *p* = 0.049), SOS (r = −0.22, *p* = 0.046), and BQI (r = −0.23, *p* = 0.034), which is not revealed by TyG–BMI (Supplementary Table S1).

Table 4 presents unadjusted and multivariable linear regression analyses assessing the association of TyG–BMI with multiple metabolic syndrome features in females. In the unadjusted analysis, all the studied variables were significantly associated with TyG–BMI. Although age (β = 1.55 [95% CI: 0.94; 2.16], *p* < 0.001) was significantly associated with TyG–BMI in the unadjusted model, its significance was lost in Model 1. For Model 1, all the core metabolic variables that were significantly associated with TyG–BMI in the univariate tests were added, particularly, age, TC, and HDL. For Model 2, mineral metabolism and bone-related parameters (Z-score, P, and VitD/PTH) were added to Model 1. HDL cholesterol, calcium, phosphorus, and the VitD/PTH ratio had a negative association in the univariate analysis.

After adjustment in Model 1, HDL cholesterol was inversely associated with TyG–BMI (β= −28.6, [95% CI: −40.5; −16.6] *p* < 0.001). A similar tendency was observed in the fully adjusted Model 2 including mineral metabolism parameters (β= −25.8 [95% CI: 33.6; −9.37]; *p* < 0.001). Although total cholesterol was an insignificant predictor in Model 1 (*p* = 0.372), in Model 2, it showed a statistically significant association (β = 6.23 [95% CI: 0.75–11.8]; *p* = 0.07). Z-score and mineral metabolism parameters were not significantly associated with TyG–BMI in the adjusted Model 2. The variance inflation factor (VIF) values were low for both the final models, showing no signs of multicollinearity.

The linear regression for males is shown in Supplementary Table S2. Age (β = 0.69, *p* = 0.057) and total cholesterol (β = 11.2, *p* = 0.034) were positively associated with TyG–BMI. Moreover, in the adjusted model, TC (β = 8.68, *p* = 0.020) was the only independent strong predictor of TyG–BMI. No associations were observed with indicators of bone health.

## 4. Discussion

In this cross-sectional study, we examined the associations between TyG-derived indices, particularly TyG–BMI, and parameters of mineral metabolism and bone quality assessed by QUS. Several key findings emerged. First, TyG-derived indices were strongly associated with classical metabolic parameters, supporting their validity as surrogate markers of insulin resistance. Second, sex-specific patterns were observed in the relationship between TyG indices and bone parameters, with more consistent associations in females than in males. Third, although certain mineral metabolism markers and derived indices showed correlations with TyG-based measures, these associations were attenuated in multivariable models. Collectively, these findings suggest a complex and potentially sex-dependent interplay between metabolic status, mineral metabolism, and bone quality.

The results of the current study show strong correlations between TyG-derived indices and triglycerides, glycemic markers, and anthropometric measures, which is consistent with previous studies. For example, a Chinese study on five TyG-derived indices found that they have a positive association with hyperuricemia: for every one standard deviation increase, the odds increased by 45–88%, and when comparing extreme quartiles, the risk was 1.61–2.29 times higher [20]. In our study, correlations of TyG–BMI with glucose, waist circumference, and HDL were strong, especially in women. A recent study that had used data from more than ninety-four thousand subjects found that high BMI and high TyG levels together increased the risk of CVD [21]. As for sex, other studies also denote that TyG–BMI has a stronger association with CVD risks in females [22]. The observed sex-specific differences may potentially be influenced by hormonal status, fat distribution, body composition, and other sex-related metabolic factors. However, detailed data on menopausal status, reproductive history, and hormone therapy were not available in the present study, and therefore these findings should be interpreted cautiously. Decreases in estrogen levels after the reproductive period result in changes in fat distribution from peripheral to central [23]. This visceral fat is metabolically active, increasing inflammation and decreasing insulin sensitivity. Additionally, after menopause, women have been shown to have increased levels of insulin resistance and less desirable lipid profiles [24]. The TyG index and related variables are adiposity measures, and therefore, it can be suggested that part of the observed gender difference is due to hormonal changes after menopause.

According to the results of this study, significant positive correlations are present between TyG–BMI and QUS parameters in females; however, no significant correlations were observed in males. These findings contribute to the ongoing discussion regarding the relationship between metabolic dysfunction and skeletal health. Studies on bone health have demonstrated that while obesity and insulin resistance are associated with higher BMD, they confer an increased risk of fractures [25]. This paradox may be due to the distinct effects of metabolic disturbances on bone mass versus bone quality. Moreover, this paradox also aligns with prior observations, as postmenopausal increases in visceral adiposity and insulin resistance may impair bone remodeling and quality, thereby compounding the adverse metabolic profile reflected by elevated TyG–BMI in women [26,27]. In addition, obesity-related limitations in mobility and balance may further increase the risk of falls, contributing to the higher fracture risk despite greater bone mass. Although TyG–BMI was associated with several bone quality parameters in correlation analyses, these associations did not remain significant after multivariable adjustment. Therefore, the present findings do not support an independent association between TyG-derived indices and skeletal parameters beyond the established metabolic factors. In this study, the incremental predictive value of TyG–BMI over its individual components was not directly evaluated and warrants further investigation.

Positive associations in females may be due, in part, to body composition influencing some bone parameters because of the higher adiposity in females resulting in greater mechanical loading and thus greater total BMD [28]. However, the findings of the current study should be interpreted with caution because the data are cross-sectional in design and are based on quantitative ultrasound measurements rather than clinical fracture outcomes. In contrast, no associations were observed in males, and inverse trends were observed in supplementary analyses examining associations with TyG–WC. The findings suggest that sex-specific metabolic determinants of bone quality were influenced by different hormonal environments, fat distribution, and muscle mass, but these factors were not investigated here. Moreover, other studies also show that males often lack similar TyG–BMI associations with bone parameters due to the androgen effect of lean mass over adiposity [29]. In contrast, the inverse association with TyG–WC was found to be associated negatively with bone parameters, possibly because of the detrimental effect of android fat mass on cortical bone quality [30].

Regarding mineral metabolism, no significant correlations were found between TyG-derived indices and mineral parameters. Exceptions were some negative correlations with indices derived from calcium and vitamin D, which were not significant after adjustment for other metabolic parameters. Limited evidence is available on the association between insulin resistance and mineral metabolism. Deficiencies in magnesium and chromium, which affect insulin action through insulin signaling and glucose uptake in skeletal muscle by the insulin-mediated translocation of GLUT-4 to the plasma membrane; a deficiency of zinc for normal beta-cell function; and even increased dietary iron, which promotes oxidative stress, can all induce insulin resistance [10,31]. However, the use of derived mineral indices to evaluate some biochemical interactions is a new approach and the results are still under investigation. Future studies are required to verify the contribution of these parameters as an independent marker of metabolic status on TyG index values.

In this study, after multivariable adjustment, in females, the strongest independent association of TyG–BMI was found with glycemic status, central adiposity, and lipid profile, while mineral and bone parameters were not independently associated with TyG–BMI. These findings support the notion that TyG-derived indices are primarily related to adiposity and glucose metabolism, and associations with bone or mineral parameters may be mediated by underlying factors that are related to both TyG and these variables [32,33]. While there are some limited mediation analyses examining the relationship between adiposity and BMD [34], there are no studies that have fully elucidated the non-linear relationship between adiposity-related indices and BMD improvement. Future research is needed to explore these complex relationships in greater detail, including longitudinal and mechanistic studies to clarify causal relationships and sex-specific effects.

### Strengths and Limitations

This study has several strengths. Firstly, it provides an integrated assessment of the metabolic status, mineral metabolism, and bone parameters simultaneously, in contrast to studies that focus solely on a few parameters and their related biological systems. Secondly, this study uses TyG-derived indices, which is a simple and practical method to assess insulin resistance using readily available clinical biochemical and anthropometric indices, including BMI. Thirdly, in addition to BMD, the assessment of total bone status by QUS provides a measure of elasticity and information regarding bone microarchitecture. Fourthly, age-adjusted partial correlation analyses were used to control for age in the analysis of the relationships between TyG–BMI and metabolic and skeletal properties of bone. Importantly, the findings of this study have importance for screening individuals at risk of metabolic disturbances in a country with a high prevalence of vitamin D deficiency, unique dietary practices, and an increasing epidemic of metabolic disturbances. The findings may provide preliminary region-specific data relevant to Central Asian populations with similar epidemiological and environmental characteristics.

However, several limitations should be noted. This study has a cross-sectional design and, therefore, no conclusions can be drawn regarding the temporal relationships or the direction of associations between variables. The results are based on data obtained from patients in a single center. We restricted the analysis to morbidly obese individuals (BMI > 40 kg/m^2^) in order to avoid confounding due to severe comorbidities as well as restrictions for anthropometric and QUS measurements in severely metabolically disturbed obese individuals. Ethnicity data were not systematically collected; therefore, potential ethnic differences in metabolic, mineral, and bone-related parameters could not be evaluated. Given the ethnic and cultural diversity of Kazakhstan, the findings should not be generalized to all Central Asian populations without caution. Therefore, the results cannot be generalized to other populations. QUS provides information on bone quality, which is complementary to BMD measurement with DXA. Therefore, the results of the present study should not be compared with those obtained with DXA. Values and indices derived from TyG were calculated using a single fasting blood sample. The intra-individual variability of triglycerides and glucose for repeat measurements was not taken into consideration. As a result, the variability may have affected the precision of the correlations, especially for the moderate strength correlations. In addition, several potential confounders, including dietary intake, physical activity, and hormonal and body composition measurements, were not evaluated and controlled for in this study. Thus, residual confounding could potentially still exist. The use of derived indices may also introduce correlations between variables that are not true confounders. However, all correlations between independent variables were within reasonable limits. Although the associations found were statistically significant, the magnitude of the associations were generally modest and may need further exploration for clinical significance. Moreover, detailed information regarding menopausal status, reproductive history, and hormone therapy was not available. In addition to the previously described limitations, the study population under investigation consisted of a preponderance of females as compared to males. As a result, the sex-specific associations identified in this investigation could have been confounded by the study population as well as by the limited statistical power to detect associations in the male subgroup. Replication of these findings in larger, multicenter studies with more balanced numbers of males and females would be helpful. Future studies should aim to be longitudinal and recruit larger, more diverse populations. Adding additional imaging and clinical fracture outcomes may also provide more insight into the observed associations.

## 5. Conclusions

In this study, TyG–BMI and TyG–WC showed significant correlations with several metabolic traits, including glycemic control and adiposity indicators for insulin resistance. Moreover, these indices also correlated with bone quality assessed by quantitative ultrasound, with sex-specific patterns. Most correlations were consistently observed in female subjects, whereas in males, these relationships were weaker or absent. In adjusted multivariable models, TyG-based metabolic indices were not independently associated with measures of mineral metabolism or bone parameters, suggesting that the relationship between metabolic risk indices and skeletal health is likely mediated by shared underlying factors. Given that TyG is a simple, low-cost, bedside index that can be calculated from readily available clinical data, it can be useful as a simple surrogate marker of metabolic status based on routinely available clinical parameters in low-resource settings.

## Figures and Tables

**Table 1:** Baseline clinical and metabolic characteristics of the study population stratified by sex (*n* = 330).

	Total (*n* = 330)	Females (*n* = 231; 70%)	Males (*n* = 99; 30%)	*p*-Value
Age (years), mean (SD)	46 (12)	46 (12)	47 (14)	0.853
*Clinical and metabolic characteristics*				
TyG–BMI, mean (SD)	247.4 (54.3)	242.2 (55.7)	260.5 (48.7)	**0.007**
TyG–WC, mean (SD)	830.8 (151.8)	795.9 (142.2)	916.3 (141.1)	**<0.001**
Triglycerides (mmol/L), mean (SD)	1.35 (0.80)	1.19 (0.65)	1.71 (0.99)	**<0.001**
LDL (mmol/L), mean (SD)	3.42 (0.96)	3.34 (0.91)	3.61 (0.96)	**0.019**
HDL (mmol/L), mean (SD)	1.26 (0.33)	1.35 (0.33)	1.05 (0.20)	**<0.001**
TC (mmol/L), mean (SD)	5.06 (0.96)	5.03 (0.96)	5.12 (0.98)	0.433
non-HDL (mmol/L), mean (SD)	3.79 (0.97)	3.70 (0.94)	4.05 (0.99)	**0.005**
Glucose (mmol/L), mean (SD)	5.48 (2.09)	5.25 (1.82)	6.04 (2.59)	**0.003**
HbA1c (%), mean (SD)	5.48 (1.79)	5.32 (1.78)	5.89 (1.80)	**0.011**
BMI (${kg/m}^{2}$), mean (SD)	28.9 (5.15)	28.8 (5.32)	29.3 (4.74)	0.402
WC (cm), mean (SD)	97.4 (13.4)	94.8 (12.7)	103.5 (12.9)	**<0.001**
HC (cm), mean (SD)	108.1 (9.76)	108.8 (9.77)	106.2 (9.55)	**0.032**

**Table 2:** Mineral metabolism parameters and bone strength indices (QUS) in the study population stratified by sex.

	Total	Females (*n* = 231; 70%)	Males (*n* = 99; 30%)	*p*-Value
*Mineral metabolism*				
Ca (mmol/L), mean (SD)	2.29 (1.22)	2.31 (1.27)	2.24 (1.16)	0.673
Mg (mmol/L), mean (SD)	0.81 (0.06)	0.80 (0.06)	0.83 (0.07)	**<0.001**
P (mmol/L), mean (SD)	2.06 (1.39)	2.17 (1.48)	1.79 (1.18)	0.365
PTH (pg/mL), mean (SD)	50.8 (20.9)	47.6 (21.8)	58.9 (20.6)	0.249
VitD (ng/mL, mean (SD)	20.8 (10.1)	20.4 (10.6)	21.7 (8.81)	0.327
Ca/Mg, mean (SD)	2.63 (0.27)	2.66 (0.24)	2.56 (0.31)	**0.005**
Ca × P, mean (SD)	17.6 (2.79)	17.8 (2.60)	17.3 (3.18)	0.125
P/Mg, mean (SD)	1.78 (0.29)	1.82 (0.28)	1.70 (0.32)	**0.002**
Ca × P/Mg, mean (SD)	9.24 (1.53)	9.40 (1.39)	8.81 (1.75)	**0.002**
VitD/PTH, mean (SD)	0.54 (0.39)	0.54 (0.39)	0.55 (0.40)	0.842
*Bone strength (QUS)*				
T-score (%), mean (SD)	77.1 (14.9)	74.5 (13.2)	83.2 (16.9)	**<0.001**
T-score, *n* (%)				**<0.001**
≤−2.5	15 (5)	13 (6)	2 (2)	
−1.0 to −2.5	199 (60)	155 (67)	44 (44)	
≥−1.0	116 (35)	63 (27)	53 (54)	
Z-score (%), mean (SD)	88.9 (18.1)	86.1 (15.9)	95.4 (21.1)	**<0.001**
Bone density by Z-score, *n* (%)				0.172
Low	35 (11)	28 (12)	7 (7)	
Normal	295 (89)	203 (88)	92 (93)	
SOS (m/s), mean (SD)	1510.9 (14.1)	1508.5 (11.4)	1516.5 (17.9)	**<0.001**
BUA (dB/MHz), mean (SD)	95.8 (22.2)	89.0 (18.2)	111.5 (24.8)	**0.024**
BQI	80.2 (15.5)	77.6 (13.7)	86.5 (17.5)	**<0.001**

**Table 3:** Age-adjusted partial correlations between TyG-derived indices and metabolic parameters stratified by sex.

	Females		Males	
	TyG–BMI	TyG–WC	TyG–BMI	TyG–WC
Triglycerides	**r = 0.51**	**r = 0.57**	**r = 0.54**	**r = 0.54**
	***p* < 0.001**	***p* < 0.001**	***p* < 0.001**	***p* < 0.001**
LDL	r = 0.09	**r = 0.14**	r = 0.07	r = 0.07
	*p* = 0.177	***p* = 0.036**	*p* = 0.505	*p* = 0.533
HDL	**r = −0.41**	**r = −0.41**	**r = −0.24**	**r = −0.32**
	***p* < 0.001**	***p* < 0.001**	***p* = 0.027**	***p* = 0.002**
TC	r = 0.01	r = 0.09	r = 0.19	r = 0.17
	*p* = 0.868	*p* = 0.203	*p* = 0.079	*p* = 0.121
Non-HDL	**r = 0.17**	**r = 0.25**	**r = 0.23**	r = 0.21
	***p* = 0.014**	***p* < 0.001**	***p* = 0.039**	*p* = 0.063
Glucose	**r = 0.54**	**r = 0.53**	r = 0.17	**r = 0.38**
	***p* < 0.001**	***p* < 0.001**	*p* = 0.111	***p* < 0.001**
HbA1c	**r = 0.44**	**r = 0.41**	r = 0.13	**r = 0.29**
	***p* < 0.001**	***p* < 0.001**	*p* = 0.230	***p* = 0.005**
HC	**r = 0.77**	**r = 0.70**	**r = 0.58**	**r = 0.62**
	***p* < 0.001**	***p* < 0.001**	***p* < 0.001**	***p* < 0.001**

**Table 4:** Linear regression analysis of factors associated with TyG–BMI in females.

	Unadjusted Model		Model 1		Model 2	
	Coef. [95% CI]	*p*-Value	Coef. [95% CI]	*p*-Value	Coef. [95% CI]	*p*-Value
Age	1.55 [0.94; 2.16]	**<0.001**	0.14 [−0.22; 0.50]	0.439	−0.11 [−0.57; 0.43]	0.913
TC	7.62 [−0.08; 15.3]	**0.052**	1.93 [−2.32; 6.17]	0.372	6.23 [0.75; 11.8]	**0.007**
HDL	−63.3 [−83.9; −42.5]	**<0.001**	−28.6 [−40.5; −16.6]	**<0.001**	−25.8 [−33.6; −9.37]	**<0.001**
Z-score	0.88 [0.42; 1.34]	**<0.001**			−0.09 [−0.31; 0.05]	0.712
P	−2.78 [−4.89; −0.67]	**0.010**			−0.21 [−2.95; 1.83]	0.851
VitD/PTH	−23.9 [−42.7; −5.19]	**0.013**			−3.70 [−14.6; 9.11]	0.572
			vif = 1.26		vif = 1.67	

## Data Availability

The data supporting the findings of this study are available from the corresponding author upon reasonable request.

## Supplementary Materials

The following supporting information can be downloaded at: https://www.mdpi.com/article/10.3390/jcm15114226/s1, Table S1: Age–adjusted partial correlations between TyG indices, mineral metabolism, and bone parameters; Table S2: Linear regression analysis of factors associated with TyG–BMI in males.

## Abbreviations

The following abbreviations are used in this manuscript:

IR	Insulin resistance
HOMA-IR	Homeostatic Model Assessment of Insulin Resistance
TyG	triglyceride–glucose
BMI	body mass index
TyG–BMI	biochemical IR and body mass index
TyG–WC	biochemical IR and waist circumference
CVD	cardiovascular diseases
VitD	Vitamin D
PTH	parathyroid hormone
BMD	bone mineral density
QUS	quantitative ultrasound
SOS	speed of sound
BUA	broadband ultrasound attenuation
BQI	bone quality index
HbA1c	Glycated hemoglobin
25(OH)D	25-hydroxy vitamin D
VIF	Variance inflation factors
