# Supplementary material for: TyG–BMI as a Marker of Metabolic Status and Its Association with Bone Quality and Mineral Metabolism: A Sex-Specific Analysis
Source: J Clin Med. 2026 May 29;15(11):4226. doi: 10.3390/jcm15114226 (PMC13257568; doi:10.3390/jcm15114226)
Supplement: Supplementary file 1 [file jcm-15-04226-s001.zip › jcm-4315447-supplementary.pdf]

Supplementary Table S1. Age–adjusted partial correlations between TyG indices, mineral metabolism, and bone parameters

	Females		Males	
	TyG–BMI	TyG–WC	TyG–BMI	TyG–WC
Ca	**r = −0.14 p=0.043**	r = −0.13 p=0.056	r = −0.09 p=0.385	r = −0.11 p=0.288
Mg	r = −0.09 p=0.163	r = −0.09 p=0.208	r = 0.04 p=0.735	r < 0.01 p=0.996
P	r = −0.13 p=0.063	r = −0.12 p=0.088	r = 0.01 p=0.939	r = −0.03 p=0.763
PTH	r = 0.08 p=0.252	r = 0.07 p=0.303	r = −0.04 p=0.704	r < 0.01 p=0.968
VitD	r = −0.12 p=0.077	r = −0.05 p=0.475	**r = −0.23 p=0.034**	r = −0.19 p=0.069
Ca/Mg	r = 0.08 p=0.272	r = 0.07 p=0.336	r = −0.08 p=0.485	r = 0.03 p=0.819
Ca × P	r = −0.11 p=0.124	r = −0.09 p=0.182	r = −0.14 p=0.196	r = −0.11 p=0.308
P/Mg	r = −0.05 p=0.434	r = −0.06 p=0.419	r = −0.14 p=0.201	r = −0.14 p=0.213
Ca × P/Mg	r = −0.06 p=0.425	r = −0.04 p=0.529	r = −0.14 p=0.209	r = −0.09 p=0.444
VitD/PTH	**r = −0.15 p=0.029**	r = −0.08 p=0.238	r = −0.13 p=0.233	r = −0.08 p=0.468
T−score (%)	**r = 0.21 p=0.002**	**r = 0.24 p<0.001**	r = −0.13 p=0.234	**r = −0.22 p=0.037**
Z−score (%)	**r = 0.21 p=0.002**	**r = 0.23 p<0.001**	r = −0.11 p=0.301	**r = −0.21 p=0.049**
SOS	**r = 0.19 p=0.007**	**r = 0.19 p=0.004**	r = −0.11 p=0.304	**r = −0.22 p=0.040**
BUA	**r = 0.18 p=0.010**	**r = 0.21 p=0.002**	r = −0.03 p=0.808	r = 0.06 p=0.611
BQI	**r = 0.21 p=0.002**	**r = 0.24 p<0.001**	r = −0.13 p=0.237	**r = −0.23 p=0.034**

Supplementary Table S2. Linear regression analysis of factors associated with TyG–BMI in males

	Unadjusted model		Model 1	
	Coef. [95% CI]	p-value	Coef. [95% CI]	*p*-value
Age	0.69 [−0.02; 1.41]	**0.057**	−0.21 [−0.75; 0.34]	0.451
TC	11.2 [0.86; 21.5]	**0.034**	8.68 [1.42; 15.9]	**0.020**
HDL	−46.8 [−97.1; 3.45]	0.068	−32.1 [−67.7; 3.48]	0.076
Glucose	3.95 [0.01; 7.88]	**0.049**	2.53 [−0.24; 5.30]	0.073

WC	2.79 [2.21; 3.37]	**<0.001**	2.71 [2.13; 3.29]	**<0.001**
Z-score	−0.08 [−0.55; 0.39]	0.745		
Ca	−1.22 [−3.66; 1.21]	0.321		
P	−0.04 [−3.26; 3.17]	0.979		
VitD/PTH	−18.3 [−45.3; 8.58]	0.179		
			vif = 1.12	
